# Dataset of a retrospective multicenter cohort study on characteristics of immune checkpoint inhibitor-induced encephalitis and comparison with HSV-1 and anti-LGI1 encephalitis

**DOI:** 10.1016/j.dib.2022.108649

**Published:** 2022-09-30

**Authors:** Leonie Müller-Jensen, Sarah Zierold, Judith M Versluis, Wolfgang Boehmerle, Petra Huehnchen, Matthias Endres, Raphael Mohr, Annette Compter, Christian U Blank, Tim Hagenacker, Friedegund Meier, Lydia Reinhardt, Anja Gesierich, Martin Salzmann, Jessica C Hassel, Selma Ugurel, Lisa Zimmer, Patricia Banks, Lavinia Spain, Jennifer A Soon, Tomohiro Enokida, Makoto Tahara, Katharina C Kähler, Ruth Seggewiss-Bernhardt, Catriona Harvey, Georgina V Long, Florian Schöberl, Louisa von Baumgarten, Thomas Hundsberger, Max Schlaak, Lars E French, Samuel Knauss, Lucie M Heinzerling

**Affiliations:** a Department of Neurology with Experimental Neurology, Charité – Universitätsmedizin Berlin, Freie Universität Berlin and Humboldt- Universität zu Berlin, Charitéplatz 1, Berlin 10117, Germany; b Berlin Institute of Health at Charité – Universitätsmedizin Berlin, Charitéplatz 1, Berlin 10117, Germany; c SERIO Side Effects Registry Immunooncology, Germany; d Department of Dermatology and Allergy, University Hospital, Ludwig-Maximilian Universität Munich, Frauenlobstr. 9-11, München 80337, Germany; e Department of Medical Oncology, Netherlands Cancer Institute, Amsterdam, the Netherlands; f NeuroCure Cluster of Excellence, Charité – Universitätsmedizin Berlin, Freie Universität Berlin, Humboldt-Universität zu Berlin, and Berlin Institute of Health, Berlin 10117, Germany; g Center for Stroke Research Berlin, Charité – Universitätsmedizin Berlin, Freie Universität Berlin, Humboldt-Universität zu Berlin, and Berlin Institute of Health, Berlin 10117, Germany; h German Center for Neurodegenerative Diseases (DZNE), Partner Site Berlin, Germany; i German Centre for Cardiovascular Research (DZHK), Partner Site Berlin, Berlin, Germany; j Department of Hepatology and Gastroenterology, Charité – Universitätsmedizin Berlin, Freie Universität Berlin and Humboldt- Universität zu Berlin, , Augustenburger Platz 1, Berlin 13353, Germany; k Department of Neuro-Oncology, Netherlands Cancer Institute, Amsterdam, the Netherlands; l Department of Medical Oncology, Division of Molecular Oncology and Immunology, Netherlands Cancer Institute, Amsterdam, the Netherlands; m Department of Neurology and Center for Translational Neuro- and Behavioral Sciences (C-TNBS), University Hospital Essen, Hufelandstr. 55, Essen 45147, Germany; n Department of Dermatology, Skin Cancer Center at the University Cancer Center Dresden and National Center for Tumor Diseases, TU Dresden, University Hospital Carl Gustav Carus, Dresden, Germany; o Department of Dermatology, Venereology and Allergology, University Hospital Würzburg, Würzburg, Germany; p Department of Dermatology and National Center for Tumor Diseases (NCT), Skin Cancer Center, University Hospital Heidelberg, Heidelberg, Germany; q Department of Dermatology, Venerology und Allergology, University Hospital Essen, Essen, Germany; r Andrew Love Cancer Centre, University Hospital Geelong, Geelong, Australia; s Medical Oncology Department, Peter MacCallum Cancer Center, Melbourne, Australia; t Department of Head and Neck Medical Oncology, National Cancer Center Hospital East, 6-5-1 Kashiwanoha, Kashiwa 277-8577, Japan; u Department of Dermatology, Venerology and Allergology, University of Schleswig-Holstein Hospital, Campus Kiel, Germany; v Department of Hematology and Oncology, Sozialstiftung Bamberg, Bamberg, Germany; w Melanoma Institute Australia, University of Sydney, Royal North Shore and Mater Hospitals, Sydney, Australia; x Department of Neurology, Ludwig-Maximilian Universität, Marchioninistraße 15, München 83177, Germany; y Division of Neuro-Oncology, Department of Neurosurgery, Ludwig-Maximilian Universität, Marchioninistraße 15, München 83177, Germany; z Department of Neurology, Kantonsspital St. Gallen, St. Gallen, Switzerland; aa Department of Dermatology, Venerology and Allergology, Charité – Universitätsmedizin Berlin, Freie Universität Berlin and Humboldt- Universität zu Berlin, Charitéplatz 1, Berlin 10117, Germany; ab Philip Frost, Department of Dermatology and Cutaneous Surgery, University of Miami Miller School of Medicine, Miami, FL, USA; ac Department of Dermatology, Friedrich-Alexander-Universität Erlangen-Nürnberg (FAU), University Hospital Erlangen, Erlangen, Germany

**Keywords:** Immunotherapy, Cancer, Immune related adverse events, Neurotoxicity, Encephalitis, Anti-LGI1 encephalitis, Herpetic encephalitis

## Abstract

Over the past decade, cancer immunotherapy with immune checkpoint inhibitors (ICIs) has significantly improved the outcome of many malignancies. However, with the broad use of ICIs, neurological immune related adverse events (irAE) are increasingly recognized. ICI-induced encephalitis (ICI-iE) is a particularly severe irAE, often leading to treatment termination, long-term sequalae or death. Despite its high morbidity and mortality, data on clinical features and diagnostic criteria are limited.

We aimed to define clinical, radiologic and laboratory characteristics of ICI-iE and identify factors that discriminate it from anti-leucine-rich glioma-inactivated (anti-LGI)-1 encephalitis and herpes simplex virus (HSV)-1 encephalitis – two alternative causes of encephalitis – to increase the awareness of ICI-iE and improve its diagnosis and management.

To that end, we retrospectively collected 30 cases of ICI-iE that were reported to the Side Effect Registry Immuno-Oncology (SERIO) and 46 cases of anti-LGI1 encephalitis or herpes simplex virus (HSV)-1 encephalitis that presented to a large German neurological referral center (Charité Universitätsmedizin Berlin) between January 2015 and September 2021. Signs and symptoms, imaging and electroencephalogram features, laboratory findings and outcome measures were assessed using standardized case report forms as well as patients’ medical records and compared between the groups.

The data reported here represents the largest primary cohort of patients with ICI-iE to date and the first comparison with other types of encephalitis. As all three disorders – ICI-iE, HSV-1 encephalitis and anti-LGI1 encephalitis – are rare neurological entities, this dataset can be used as a reference in future clinical studies on ICI-induced neurotoxicity, neurological autoimmune disorders, and central nervous system infections.

**Specifications Table**

**Table:** 

Subject	Health and medical sciences: Clinical Neurology
Specific subject area	Clinical and diagnostic data on immune-checkpoint inhibitor-induced encephalitis
Type of data	Tables
How the data were acquired	Data were collected using standardized case report forms and patient's electronic medical records.
Data format	Raw Analyzed
Description of data collection	We screened the Side Effect Registry Immuno-Oncology (SERIO) and queried cooperating cancer centers in Germany, the Netherlands, Japan, and Australia for cases of ICI-iE. To identify cases of HSV-1 or anti-LGI1 encephalitis we searched the electronic medical records of a large German neurological referral center (Charité Universitätsmedizin Berlin). We evaluated all consecutive patients presenting between January 2015 and September 2021.
Data source location	Institution: Charité - Universitätsmedizin Berlin City/Town/Region: Berlin Country: Germany
Data accessibility	Repository name: Mendeley Data Data identification number: DOI: 10.17632/vfpxcbk5bx.3 Direct URL to data:https://data.mendeley.com/datasets/vfpxcbk5bx
Related research article	L. Müller-Jensen, S. Zierold, J.M. Versluis, W. Boehmerle, P. Huehnchen, M. Endres, R. Mohr, A. Compter, C.U. Blank, T. Hagenacker, F. Meier, L. Reinhardt, A. Gesierich, M. Salzmann, J.C. Hassel, S. Ugurel, L. Zimmer, P. Banks, L. Spain, J.A. Soon, T. Enokida, M. Tahara, K.C. Kähler, R. Seggewiss-Bernhardt, C. Harvey, G. v. Long, F. Schöberl, L. von Baumgarten, T. Hundsberger, M. Schlaak, L.E. French, S. Knauss, L.M. Heinzerling, Characteristics of immune checkpoint inhibitor-induced encephalitis and comparison with HSV-1 and anti-LGI1 encephalitis: A retrospective multicentre cohort study, Eur J Cancer. 175 (2022) 224–235. https://doi.org/10.1016/J.EJCA.2022.08.009.

## Value of the Data

• These data give new insight into the clinical characteristics and diagnostic features of immune checkpoint inhibitor-induced encephalitis (ICI-iE), a rare but increasingly recognized toxicity of ICI treatment.
• The comparison to other types of encephalitis can improve the diagnostic accuracy of ICI-iE, providing valuable information for clinicians and researchers.
• This dataset can be used as a reference for future clinical studies on ICI-induced neurotoxicity, neurological autoimmune disorders, and central nervous system infections.

## 1 Objective

Until now, data on clinical features, diagnostic criteria and treatment of irEncephalitis have been limited to case reports and series with small sample sizes. Data to describe clinical differentiators from other types of encephalitis is largely missing. However, patients with extensive malignant disease are also at risk of infectious encephalitis, such as herpes simplex virus (HSV)-1 encephalitis. The objective of this dataset is to provide data on clinical, laboratory, and radiologic features of irencephalitis and allow for comparison with herpes simplex virus (hsv)-1 encephalitis and anti-leucine-rich glioma-inactivated (anti-lgi)-1 encephalitis in support of the related research article.

## 2 Data Description

Analyzed demographics and clinical data of patients with ICI-iE, HSV-1 encephalitis and anti-LGI1 encephalitis included in this dataset are published in the related research article [1].

*ICI-iE_raw_data.xlsx* provides the standardized case report form with raw data of all screened patients with ICI-iE and all included patients with HSV-1 encephalitis and anti-LGI1 encephalitis. For patients with ICI-iE the investigators of the respective cancer centers completed the case report form. For patients with HSV-1 or anti-LGI1 encephalitis two authors (LMJ and SK) extracted data from patient's electronic medical records and then completed the case report form. Missing values are reported as “NA” (= not available).

*ICI-iE_Table_1.pdf* provides raw data of patients with ICI-iE that were included for further analysis. In contrast to *ICI-IE_raw_data, ICI-iE_Table_1* is a formatted table that contains a selection of important clinical and diagnostic data. In addition, it reports the diagnostic certainty according to the consensus disease definition by Guidon et al. [1].

*ICI-iE _Table_2.pdf* provides raw data of patients with suspected ICI-iE that were excluded from further analysis. In contrast to *ICI-IE_raw_data, ICI-iE _Table_2* is a formatted table that contains a selection of important clinical and diagnostic data. In addition, it reports the diagnostic certainty according to the consensus disease definition by Guidon et al. [1].

*ICI-iE_p_values.xlsx* provides all p-values and the correlation coefficient (rho) that were calculated. Each row represents one test.

## 3 Experimental Design, Materials and Methods

### 3.1 Patient Selection and Data Collection

We collected cases of ICI-iE, HSV-1 encephalitis and anti-LG1 encephalitis between January 2015 and September 2021.

We screened all consecutive patients with suspected ICI-iE that were reported to Side Effect Registry Immuno-Oncology (SERIO), an international registry based at the Ludwig-Maximilian University Hospital in Munich that collects cases of irAE in cooperation with the Paul Ehrlich Institute. Furthermore, we queried cancer centers in Germany, Japan, Australia and the Netherlands for additional cases. For our control groups we screened the electronic medical records of the Charité Universitätsmedizin Berlin for patients with HSV-1 encephalitis and anti-LGI1 encephalitis using the ICD-10 diagnoses B00.4 (herpetic encephalitis) and G.04.x (encephalitis, myelitis, and encephalomyelitis) as search terms.

Using a standardized case report form the investigators of the respective cancer centers or *LMJ* and *SK* (for patients with HSV-1 or anti-LGI1 encephalitis) collected the following data: Demographics, tumor entity, ICI type, additional irAEs, CTCAE grade of ICI-iE, symptoms at onset, findings in the neurological exam, comorbidities, results of brain MRI and EEG, CSF analyses, blood tests (white blood cell count [WBC], C-reactive protein [CRP] and procalcitonin [PCT]), modified Rankin Scale (mRS) score, treatment, and clinical outcome. Outcome was defined as full recovery (= no signs of encephalitis), recovery with sequelae (= residual signs of encephalitis) or ongoing symptoms, and death. Symptoms at presentation were assessed as an open-ended item and as a query (yes / no) on the following predefined signs and symptoms: impaired consciousness, memory deficits, disorientation, confusion, behavioral and personality changes, headache, fever, seizures, hallucinations, hyponatremia, aphasia, signs of cerebellar dysfunction, other focal deficits, and meningism.

### 3.2 Disease Definitions

We included patients with ICI-iE that met consensus criteria of *definite, probable,* or *possible* ICI-iE, described previously by Guidon et al. Cases of *possible* ICI-iE were only included if patients responded to immunosuppressive treatment and the course of the disease made alternative diagnoses improbable. Exclusion of alternative causes included brain MRI in all patients and CSF examination (including culture and PCR-screening for common pathogens).

HSV-1 encephalitis was defined as acute onset of (meningo-)encephalitis with typical signs (e.g., fever, seizures, confusion) [2] plus positive HSV-1-PCR in CSF or positive HSV-1-specific serum/CSF antibody ratio at hospitalization. Anti-LGI1 encephalitis was defined as (sub)acute onset of limbic encephalitis with typical signs of anti-LGI1 encephalitis [3,4] and positive anti-LGI1 antibodies in serum and/or CSF tested with indirect immunofluorescence assays. CT or MRI was obligatory to exclude alternative pathologies. We only included patients with HSV-1 or anti-LGI1 encephalitis that had not been treated with ICIs.

Two investigators (LMJ and SK) confirmed the diagnosis of ICI-iE, HSV-1 and anti-LGI1 encephalitis according to the criteria above. If *LMJ* and *SK* disagreed, the case was discussed with a third investigator (*PH*) until consensus was achieved.

### 3.3 Statistical Analyses

Categorical variables are presented as observed counts and percentages. Continuous variables are presented as median and interquartile range (IQR). We used pairwise deletion to handle missing data. Missing data are indicated as “NA”. Group comparisons of categorical data were performed using Chi-squared test or Fisher's exact test (the latter if >20% of cells had expected frequencies <5). Group comparisons of continuous data were performed using the Kruskal–Wallis test. We used the two-sided Spearman's correlation coefficient for our correlation analysis and calculated 95% bootstrap confidence intervals. *P* values ≤ 0.05 were considered statistically significant. We corrected for multiple comparisons using the false discovery rate method [5]. We conducted statistical analyses and graphic illustrations using IBM SPSS Statistics software (version 27.0), Microsoft Excel (version 16.52), Graph Pad Prism (version 7).

## Ethics Statements

The institutional review board in Erlangen (exemption dating 03.09.2012; 17_16Bc, 29.01.2016; 2_20B and 05.05.2020) and Munich (dating 17.2.2021; 20-1122) approved data analysis from the SERIO registry. At the Charité Universitätsmedizin Berlin, §25 of the Berlin legislation for hospitals allows the use of routine care data for scientific purposes. For patients enrolled at other cancer centers, each respective center had approval or exemption of the local ethics committee. This study was conducted in accordance with the Declaration of Helsinki.

## CRediT authorship contribution statement

**Leonie Müller-Jensen:** Conceptualization, Methodology, Investigation, Formal analysis, Writing – original draft, Visualization. **Sarah Zierold:** Investigation, Writing – review & editing. **Judith M Versluis:** Investigation, Writing – review & editing. **Wolfgang Boehmerle:** Conceptualization, Methodology, Supervision, Writing – review & editing. **Petra Huehnchen:** Conceptualization, Methodology, Supervision, Writing – review & editing. **Matthias Endres:** Supervision, Writing – review & editing. **Raphael Mohr:** Investigation, Writing – review & editing. **Annette Compter:** Investigation, Writing – review & editing. **Christian U Blank:** Investigation, Writing – review & editing. **Tim Hagenacker:** Investigation, Writing – review & editing. **Friedegund Meier:** Investigation, Writing – review & editing. **Lydia Reinhardt:** Investigation, Writing – review & editing. **Anja Gesierich:** Investigation, Writing – review & editing. **Martin Salzmann:** Investigation, Writing – review & editing. **Jessica C Hassel:** Investigation, Writing – review & editing. **Selma Ugurel:** Investigation, Writing – review & editing. **Lisa Zimmer:** Investigation, Writing – review & editing. **Patricia Banks:** Investigation, Writing – review & editing. **Lavinia Spain:** Investigation, Writing – review & editing. **Jennifer A Soon:** Investigation, Writing – review & editing. **Tomohiro Enokida:** Investigation, Writing – review & editing. **Makoto Tahara:** Investigation, Writing – review & editing. **Katharina C Kähler:** Investigation, Writing – review & editing. **Ruth Seggewiss-Bernhardt:** Investigation, Writing – review & editing. **Catriona Harvey:** Investigation, Writing – review & editing. **Georgina V Long:** Investigation, Writing – review & editing. **Florian Schöberl:** Investigation, Writing – review & editing. **Louisa von Baumgarten:** Investigation, Writing – review & editing. **Thomas Hundsberger:** Investigation, Writing – review & editing. **Max Schlaak:** Investigation, Writing – review & editing. **Lars E French:** Investigation, Writing – review & editing. **Samuel Knauss:** Conceptualization, Methodology, Formal analysis, Writing – review & editing. **Lucie M Heinzerling:** Conceptualization, Investigation, Funding acquisition, Supervision, Writing – review & editing.

## Declaration of Competing Interest

The authors declare that they have no known competing financial interests or personal relationships that could have appeared to influence the work reported in this paper.

X The authors declare the following financial interests/personal relationships which may be considered as potential competing interests: LMJ, JMV, WB, PH, RM, AC, TH, FM, LR, PB, JAS, TE, MT, CH, LvB, THu, declare no conflicts of interest. SZ declares speaker's fees or travel grants from Bristol-Myers Squibb (BMS), Sun Pharma and Merck Sharp & Dohme (MSD). ME received funding from DFG under Germany´s Excellence Strategy – EXC-2049 – 390688087, BMBF, DZNE, DZHK, EU, Corona Foundation, and Fondation Leducq. ME reports grants from Bayer and fees paid to the Charité from AstraZeneca, Bayer, Boehringer Ingelheim, BMS, Daiichi Sankyo, Amgen, GSK, Sanofi, Covidien, Novartis, Pfizer, all outside the submitted work. CUB received compensation (all paid to the institute except TRV) for advisory roles for BMS, MSD, Roche, Novartis, GlaxoSmithKline, AstraZeneca, Pfizer, Lilly, GenMab, Pierre Fabre, Third Rock Ventures, received research funding (all paid to the institute) from BMS, Novartis, NanoString, and declares stockownership in Immagene BV, where he is co-founder. AG received speaker´s honoraria from Allmiral, BMS, MSD and Roche; AG has intermittent advisory board relationships with Amgen, BMS, Novartis, MSD, Pierre Fabre Pharmaceuticals, Pfizer, Roche and Sanofi Genzyme; travel and congress fee support from BMS, MSD, Novartis, Pierre Fabre Pharmaceuticals and Roche. Clinical studies: Amgen, Array, BMS, GSK, Novartis, Merck, MSD, Pfizer, and Roche. MS received speaker's honoraria and travel grants from Abbvie, BMS, Merck, MSD, Novartis, Pfizer, and Sanofi. JCH has served as a consultant for GSK, MSD, Pierre Fabre, Sun Pharma (personal) and BMS, Immunocore, Nektar, Novartis, Philogen (institution); received speaker's honoraria from Almirall, Amgen, BMS, GSK, MSD, Novartis, Pfizer, Pierre Fabre, Roche, Sanofi (personal); a scientific grant from BMS and Sun Pharma (institution) and clinical trial honoraria for BioNTech, BMS, Genentech, Immunocore, Iovance, MSD, Novartis, Philogen, Pierre Fabre, Regeneron, Roche, Sanofi, 4SC (institution). SU declares research support from BMS and Merck Serono; speakers and advisory board honoraria from BMS, MSD, Merck Serono, Novartis and Roche, and travel support from BMS, MSD, and Pierre Fabre Pharmaceuticals. LZ served as consultant and/or has received honoraria from BMS, MSD, Novartis, Pierre Fabre Pharmaceuticals, Sun Pharma and Sanofi; Research funding to institution: Novartis; travel support from MSD, BMS, Amgen, Pierre-Fabre, Sun Pharma, Sanofi and Novartis, outside the submitted work. LS declares advisory and speaker honoraria from BMS. KCK has served as consultant or/and has received honoraria from Amgen, Roche, BMS, MSD, Pierre Fabre Pharmaceuticals, and Novartis, and received travel support from Amgen, BMS, MSD, Amgen, Pierre Fabre Pharmaceuticals, Medac and Novartis. RSB has served as consultant for and/or received honoraria from Amgen, AstraZeneca, BMS, Celgene, Jansen-Cilag, MSD, Merck Seono, Novartis, Pfizer, and Roche. GVL is consultant advisor for Agenus, Amgen, Array Biopharma, Boehringer Ingelheim, BMS, Evaxion, Hexal AG (Sandoz Company), Highlight Therapeutics S.L., Innovent Biologics USA, MSD, Novartis Pharma AG, OncoSec, PHMR Limited, Pierre Fabre Pharmaceuticals, Provectus, Qbiotics, Regeneron. FS has received an honorarium from Gilead for an advisory board meeting. MS received speaker's honoraria and participated in advisory boards of BMS, Novartis, MSD, Roche, Pierre Fabre Pharmaceuticals, Kyowa Kirin, Immunocore, Recordati and Sanofi-Genzyme. MS received travel accommodation and expenses by Novartis, Pierre Fabre Pharmaceuticals, and Sun Pharma. LEF has served as consultant for Galderma, Janssen, Leo Pharma, Eli Lilly, Almirall, Union Therapeutics, Regeneron, Novartis, Amgen, Abbvie, UCB, Biotest, and InflaRx. SK received compensation for advising roles for Biogen. LH has served as consultant for Amgen, BMS, Biome-dx, EMA, Immunocore, Kyowa Kirin, MSD, Novartis, Pierre Fabre Pharmaceuticals, Roche, and Sanofi.

## Data Availability

Dataset of a Retrospective Multicenter Cohort Study on Characteristics of Immune Checkpoint Inhibitor-induced Encephalitis and Comparison with HSV-1 and Anti-LGI1 Encephalitis (Original data) (Mendeley Data).
